# William Fiero Eddington

**Published:** 1883-02

**Authors:** 


### OBITUARY.

---

*William Fiero Edington. M. D., D. D. S*, died at his residence in Geneva, New York, on Saturday, January 27th 1883. Dr. Edington had been ill for more than two years, but not seriously until some two weeks before his death, his affection being Brights' disease of the kidneys, complicated with organic disease of the heart.

Dr. Edington was born in the present town of Seneca, as were his parents. He was born in February 17, 1826, being nearly 57 years of age at the time of his death. He studied dentistry, and graduated at the Baltimore College of Dental Surgery, of the class of 1856, immediately after practicing at Dundee, N. Y. In 1854 he removed to Geneva, continuing his practice. He then studied medicine and received his degree of M. D. degree of M. D. from, the Geneva Medical College.

Dr. Edington was an honorable and spirited citizen, and occupied an exalted reputation as a dental practitioner. He was a warm friend of the Dental Department of the University of Maryland, being a member of the corps of Clinical Instructors of that institution at the time of his death, and always manifested a great interest in dental education.

